# A systematic review of the patient burden of Crohn’s disease-related rectovaginal and anovaginal fistulas

**DOI:** 10.1186/s12876-021-02079-8

**Published:** 2022-01-28

**Authors:** Kristy Iglay, Dimitri Bennett, Michael D. Kappelman, Sydney Thai, Molly Aldridge, Chitra Karki, Suzanne F. Cook

**Affiliations:** 1 CERobs Consultancy LLC, Wrightsville Beach, NC 27480 USA; 2 Gillings School of Public Health, Epidemiology, University of North Carolina-Chapel Hill, Chapel Hill, NC 27599 USA; 3 Takeda Pharmaceuticals, Cambridge, MA 02139 USA; 4 Perelman School of Medicine, University of Pennsylvania, Philadelphia, PA 19104 USA; 5 Chapel Hill School of Medicine, Pediatric Gastroenterology, University of North Carolina, Chapel Hill, NC 27599 USA

**Keywords:** Crohn’s disease, Anovaginal fistula, Rectovaginal fistula, Epidemiology, Treatment patterns, Disease burden

## Abstract

**Background:**

Crohn’s disease (CD)-related rectovaginal fistulas (RVFs) and anovaginal fistulas (AVFs) are rare, debilitating conditions that present a substantial disease and treatment burden for women. This systematic literature review (SLR) assessed the burden of Crohn’s-related RVF and AVF, summarizing evidence from observational studies and highlighting knowledge gaps.

**Methods:**

This SLR identified articles in PubMed and Embase that provide data and insight into the patient experience and disease burden of Crohn’s-related RVF and AVF. Two trained reviewers used pre-specified eligibility criteria to identify studies for inclusion and evaluate risk of bias using the Risk Of Bias In Non-randomized Studies of Interventions (ROBINS-I) tool for observational studies.

**Results:**

Of the 582 records identified, 316 full-text articles were assessed, and 16 studies met a priori eligibility criteria and were included. Few epidemiology studies were identified, with one study estimating the prevalence of RVF to be 2.3% in females with Crohn’s disease. Seven of 12 treatment pattern studies reported that patients had or required additional procedures before and/or after the intervention of interest, demonstrating a substantial treatment burden. Seven of 11 studies assessing clinical outcomes reported fistula healing rates between 50 and 75%, with varying estimates based on population and intervention.

**Conclusions:**

This SLR reports the high disease and treatment burden of Crohn’s-related RVF and AVF and identifies multiple evidence gaps in this field. The literature lacks robust, generalizable data, and demonstrates a compelling need for substantial, novel research into these rare and debilitating sequelae of CD.

*Registration* The PROSPERO registration number for the protocol for this systematic literature review is CRD42020177732.

**Supplementary Information:**

The online version contains supplementary material available at 10.1186/s12876-021-02079-8.

## Background

An estimated 780,000 people in the USA are living with Crohn’s disease (CD), and the incidence of the condition is increasing rapidly worldwide [1, 2]. In addition to the debilitating effects of intestinal inflammation, approximately 35% of patients with CD develop fistulas [3]—abnormal connections between the gastrointestinal tract and an epithelial-lined surface such as skin (external or cutaneous fistulas) or internal organs; the peritoneal space, retroperitoneal areas, or the thorax (internal fistulas) [4]. Fistulas involving the vagina, such as rectovaginal fistulas (RVFs) and anovaginal fistulas (AVFs), can be particularly upsetting and embarrassing for women, with the most common symptoms including passage of gas and/or stool via the vagina [5, 6].

Fistulizing CD is characterized by variable clinical presentations [3], but patients with RVF almost always have severe discomfort and pain, and may suffer psychological effects, including anxiety or poor body image due to malodorous drainage fluid [4, 6, 7]. For many, the natural history includes frequent recurrence after treatment and long episodes of actively draining fistulas [3]. Treatment for fistulas can be complex, including multiple pharmacological and surgical treatments, hospitalizations, and medical visits that result in high healthcare costs [6–11].

Despite RVF and AVF being debilitating conditions, there is limited observational literature on the epidemiology and disease burden of these conditions. We performed a systematic literature review (SLR) to summarize available information and highlight knowledge gaps regarding the disease burden for patients with CD-related RVF and AVF. The objective of this SLR was to identify evidence relating to incidence and prevalence, pharmacological and surgical interventions, pre-specified clinical outcomes (e.g., healing and response rates), patient-reported outcomes (PROs), and healthcare resource utilization (HCRU) including costs in observational studies of populations with RVF and AVF.

## Methods

### Search strategy and selection criteria

The SLR was conducted in accordance with the Preferred Reporting Items for Systematic Reviews and Meta-Analyses (PRISMA) [12]. The protocol for this study was registered with the International Prospective Register of Systematic Reviews (PROSPERO; registration number CRD42020177732).

Eligibility criteria were organized around the elements of Population, Intervention, Comparison, Outcomes, Time, and Study Design (PICOTS) and are included in Additional file 1: Table S1. The electronic search was conducted on March 25, 2020, utilizing a search strategy incorporating terms for fistula type (RVF and AVF), study design, and outcomes of interest in PubMed and Embase (Additional file 1: Table S2 and Additional file 1: Table S3). Search results were filtered to include only English language (PubMed, Embase) and human studies (PubMed), and limit the publication time frame to 10 years prior to the search date. To ensure relevant studies were not missed in the electronic search, a manual search of key publications and references was conducted.

The titles and abstracts for studies identified through the search were independently screened by two reviewers trained in epidemiology and the conduct of SLRs in order to determine whether they met the PICOTS criteria. Studies meeting the PICOTS criteria were carried forward to the full-text review phase, where the full-text articles were independently assessed by each reviewer to determine final eligibility for data abstraction. Any discrepancies in either step were resolved by consensus, and if consensus could not be achieved, a third trained reviewer made the determination. Data from each eligible study were independently abstracted by two reviewers using a standardized data abstraction form. Both reviewers then jointly examined the abstraction spreadsheets in order to synthesize the abstracted data into one master spreadsheet. Data were extracted for a range of variables, including study type and design, population, outcomes, and limitations.

Studies included in this article met the following criteria: (1) reported on RVF or AVF, (2) used an observational study design (i.e., case–control, cohort/registry, or cross-sectional study), (3) measured one of the outcomes of interest—incidence/prevalence, treatment patterns, clinical outcomes (healing/failure/recurrence rates, post-operative infection), PROs (Crohn’s Disease Activity Index [CDAI], Inflammatory Bowel Disease Questionnaire [IBDQ], Perianal Disease Activity Index [PDAI], 5-dimensional EuroQoL questionnaire [EQ-5D], fecal incontinence, pain, discharge/soiling, pad use, alterations in or dissatisfaction with sexual intercourse/activity), and HCRU/costs, and (4) were original research (Additional file 1: Table S1). Case series were designated as cohort studies only if they met all of the following pre-specified criteria: > 10 patients per fistula type, patients sampled based on their exposure only (not outcome), outcome assessed over a pre-specified follow-up period or mean/median follow-up reported, information available to calculate the absolute/relative risk. In addition, sampling had to be labeled as ‘consecutive’ or text had to indicate that all eligible patients were included to avoid selection of unique cases. Further, AVF and RVF were to be reported separately from other types of fistula.

### Risk of bias assessment

Two independent reviewers evaluated risk of bias in each article using the Risk Of Bias In Non-randomized Studies of Interventions (ROBINS-I) tool for observational studies [13]. The ROBINS-I tool allows investigators to rate the risk of bias in non-randomized studies in a systematic way through the evaluation of seven pre-specified domains of bias (confounding, selection of participants, classification of interventions, deviations from intended interventions, missing data, measurement of outcomes, and selection of reported result). Any disagreements were settled by consensus. If consensus could not be achieved, a third trained reviewer made the final determination.

## Results

### Systematic literature review

**Fig. 1:**
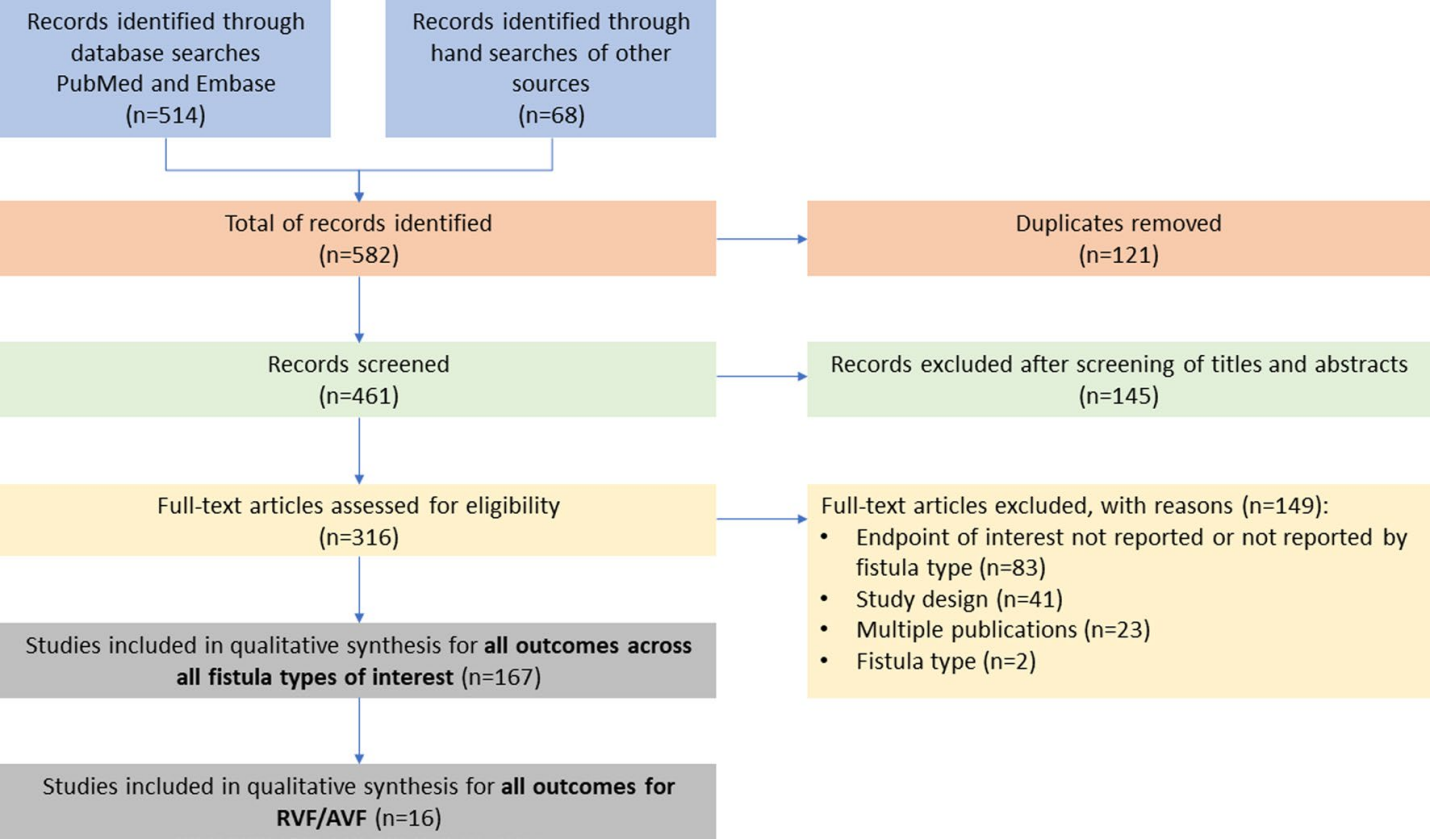
PRISMA flow diagram. *AVF* anovaginal fistula*, PRISMA* Preferred Reporting Items for Systematic Reviews and Meta-Analyses, *RVF* rectovaginal fistula

Figure 1 displays the PRISMA diagram. The electronic search returned 514 articles; an additional 68 were identified from a manual search of other sources; 121 duplicates were deleted. Of the 461 records screened based on their titles and abstracts, 316 were included in the full-text assessment. Of those, 149 were excluded based on the inclusion/exclusion criteria (Fig. 1). A total of 16 studies were included in the qualitative synthesis for RVF and AVF: 14 addressing RVF only, 1 addressing AVF only, and 1 addressing both RVF and AVF (combined) (Table 1).

**Table 1:** Characteristics of papers included in the SLR (*n* = 16 studies)

Author, year	Country, location	Inclusion criteria	Exclusion criteria	Fistula type	Sample size	Intervention(s)	Risk of bias (ROBINS-I)
Corte, 2015 [9]	France, Beaujon Hospital, Paris	Women undergoing surgery for RVF (1996–2014), includes multiple etiologies (CD, post-operative, obstetrical, post-radiation, pelvic cancer, diverticulitis, trauma, unknown)	Not reported	RVF	79 RVFs	Conservative procedures: seton drainage, vaginal advancement flap, rectal advancement flap, diverting stoma only, fistula plug, fibrin glue Major procedures: GMT, biomesh interposition, standard CAA or CRA, delayed CAA, abdominoperineal excision	Moderate
El-Gazzaz, 2010 [14]	USA^a^	Women with CD-related RVF who underwent surgical repair with intent to close the fistula from 1997 to 2007	Surgical procedures not intended for fistula closure (e.g., seton placement, diverting stoma alone, or definitive proctectomy without reconstruction)	RVF	65 RVFs	Advancement flap, CAA, episioproctotomy, fibrin glue, plug	Moderate (ClinRO) Serious (PRO)
Gaertner, 2011 [15]	USA^b^	Women with CD who underwent operative treatment for RVF between March 1998 and December 2005	Perianal fistula	RVF	51 RVFs	Operative treatment, operative treatment + infliximab	Moderate
Göttgens, 2017 [27]	Netherlands, IBDSL registry	Women with CD diagnosed January 1991–July 2011 at age ≥ 18 years	Not reported	RVF	17 RVFs	N/A	Low
Haennig, 2015 [10]	France, gastroenterology department, Hôpital Rangueil, Toulouse	Women with a perianal CD anorectal or vaginal fistula referred between 2000 and 2010	Patients with follow-up < 6 months or with enteric fistula or ECF	RVF	12 RVF	Seton drainage and associated treatment, infliximab, external drainage, fibrin glue, advancement flap, fistulotomy Other treatments (external drainage + infliximab, fistulotomy + infliximab, advancement flap + infliximab, infliximab [monotherapy], external drainage, bowel diversion)	Moderate
Jarrar, 2011 [16]	USA, Department of Colorectal Surgery, Digestive Diseases Institute, Cleveland Clinic Foundation, Cleveland, OH	Women who underwent transanal endorectal advancement flap repair of complex^c^ anal fistula by the same surgeon from 1995 to 2005	Patients with subcutaneous and superficial trans-sphincteric fistulas treated with fistulotomy alone or fistulotomy and a cutting seton	AVF	21 AVFs	Transanal endorectal advancement flap repair	Moderate
Korsun, 2019 [17]	Germany, surgery departments of the University Hospital Regensburg and the St Josef Hospital Regensburg	Women with AVF, RVF, rectourethral fistulas, or pouch-vaginal fistulas and diagnosed with IBD who underwent GMT or re-transposition for a recurrent fistula between January 2000 and May 2018	Patients with IBD who underwent GMT strictly owing to fecal incontinence and not for fistula treatment	RVF	21 RVFs 2 AVFs	GMT	Moderate
Manne, 2016 [26]	USA, Department of Medicine—Gastroenterology, University of Alabama at Birmingham, Alabama	Women with CD who underwent RVF surgery (either mucosal flap surgery or seton placement) between 2000 and 2013 for whom key demographic and medical history data were available	Not reported	RVF	16 surgeries	Mucosal flap procedure, seton	Critical
Milito, 2019 [18]	Italy, University Hospital of Tor Vergata, Rome	Women with CD who underwent surgery for an RVF performed by the same senior surgeon at a tertiary center	Not reported	RVF	43 RVFs	Surgical procedures for RVF (surgical approaches included drainage and seton, rectal advancement flap, vaginal advancement flap, transperineal approach using porcine dermal matrix, and Martius flap)	No information
Narang, 2016 [19]	USA^d^	All women who underwent RVF repair from July 1997 to June 2013 at two major tertiary referral centers Women who had recurrent symptoms at the time of the telephone survey but who had not visited their surgeon for full evaluation	Patients who did not agree to participate in the telephone follow-up survey or could not be reached	RVF	99 RVFs	Episioproctotomy, muscle interposition (including GMT and Martius flap), placement of biological plug and fibrin glue, rectal-advancement flap, sphincteroplasty, and transvaginal repair	Serious
Oakley, 2015 [20]	USA^e^	Possible cases of RVFs identified using ICD-9 codes of female genital digestive tract fistula July 2006–June 2011. Outpatient records with relevant ICD codes^f^	Charts with missing data for diagnosis or management	RVF	106 RVFs 50 AVFs 20 unspecified RVFs or AVFs	N/A	Serious
Park, 2019 [21]	USA, Olmsted Medical Center, Mayo Medical Center,Rochester MN	Women diagnosed with CD from 1970 to 2010	Not reported	RVF AVF	13 RVFs or AVFs	N/A	Low
Pinto, 2010 [22]	USA^g^	Women who underwent RVF repairs from January 1988 to May 2008 and who were surgically treated for AVFs and pouch vaginal fistulas	Patients with a rectourethral or anoperineal fistula; treated with only a diverting stoma; had < 3 months’ follow-up time; had a history of proctectomy or Hartmann procedure	RVF	45 of 125 RVFs were CD related	Endorectal advancement flap, GMT, transvaginal approach, transperineal approach	Moderate
Sapci, 2019 [23]	USA, surgical center not specified	Women diagnosed with CD who underwent surgery for RVF between 2010 and 2017	Surgery without intent to close the fistula; < 6 months’ follow-up; inadequate follow-up to verify fistula status	RVF	19 RVFs	Procedures to definitively close RVF: transanal advancement flap, transanal repair with tissue interposition (Martius or gracilis flap), episioproctotomy, fistulotomy, CAA, fistula plug	Moderate
Schloericke, 2017 [24]	Germany, Department of Surgery, University of Schleswig–Holstein, Campus Luebeck and Department of Surgery, WKK Heide	Women who underwent treatment for AVF or RVF in the period January 2000 to September 2016	Not reported	RVF	58 RVFs	Non-resective procedures (transrectal/transvaginal omentoplasty or closure); resective procedures (low anterior resection, subtotal colectomy, proctectomy, pelvic exenteration, double-barrel sigmoidostomy)	Moderate
Schwartz, 2019 [25]	USA	Cases of CD (≤ 1 claim of CD-related ICD-9 code in recent 5-year history) identified through December 31, 2014 with codes for fistulizing disease (identified by ICD-9 and surgical codes) in the Truven Health MarketScan database	Not reported	RVF	N/A	N/A	Moderate

*AVF* anovaginal fistulas, *CAA* coloanal anastomosis, *CD* Crohn’s disease, *ClinRO* clinician-reported outcome, *CRA* colorectal anastomosis, *ECF* entero-cutaneous fistula, *GMT* gracilis muscle transposition, *IBD* inflammatory bowel disease, *IBDSL* Inflammatory Bowel Disease South Limburg Cohort, *ICD* International Classification of Diseases, *ICD-9* International Classification of Diseases, ninth revision, *N/A* not applicable, *PRO* patient-reported outcome, *ROBINS-I* Risk Of Bias In Non-randomised Studies of Interventions, *RVF* rectovaginal fistula, *SLR* systematic literature review

^a^Surgical center not specified, but authors affiliated with Department of Colorectal Surgery, Cleveland Clinic, Cleveland, OH

^b^Surgical center not specified, but authors affiliated with Division of Colon and Rectal Surgery, Department of Surgery, University of Minnesota, Minneapolis, MN

^c^Complex fistulas were defined as deep trans-sphincteric fistulas, fistulas with extensions of the primary track or associated abscess, fistulas associated with CD, anovaginal fistulas, and horseshoe fistulas

^d^Department of Colorectal Surgery, Cleveland Clinic Florida, Weston, FL and Department of Colorectal Surgery, Cleveland Clinic, Cleveland, OH

^e^Twelve academic sites affiliated with female pelvic medicine and reconstructive surgery fellowship programs in the USA

^f^Selected ICD codes included 565.1 (fistula, anal); 596.1 (intestine-vesical fistula); 596.2 (vesical fistula, not elsewhere classified); 619.0 (urinary-genital tract fistula, female); 619.1 (digestive-genital tract fistula, female); 619.2 (genital tract-skin fistula, female); 619.8 (other specified fistula involving female genital tract); 619.9 (unspecified fistula involving female genital tract)

^g^Surgical center not specified, but authors affiliated with the Department of Colorectal Surgery, Cleveland Clinic Florida, Weston, FL and Department of Colorectal Surgery, Cleveland Clinic, Cleveland, OH

Most studies (n = 14) were retrospective cohort studies or case series that met the review definition for cohort studies [9, 10, 14–25]. The SLR also included one retrospective, unmatched case–control study [26] and one prospective cohort/registry study [27]. Two of the retrospective studies [14, 19] included prospective follow-up data collection (e.g., via telephone). All but two of the included reports [25, 27] came from either single- or multi-site, clinic-based studies, most often from surgical centers.

Two of the papers were identified as having a low risk of bias, and 10 papers had a moderate risk of bias. Serious or critical risk of bias was identified in three papers [19, 20, 26]. Risk of bias could not be determined in one source owing to lack of information in a published poster abstract [18] (Table 1).

### Incidence and prevalence

Three of the 16 included papers provided population-based estimates of RVF incidence or prevalence (Table 2). A study of a population-based inflammatory bowel disease cohort in the well-defined South Limburg area of the Netherlands from 1991‒2014 reported that 17 of 728 female patients with CD (2.3%) had RVF [27]. A large, claims-based study conducted in the Truven Health MarketScan database by Schwartz et al. (2019) identified cases of CD with codes for fistulizing disease and estimated that > 6000 women were affected by RVF in the USA [25]. It should be noted that this study had a limited follow-up period and used International Classification of Diseases codes that may have poor validity in this area.

**Table 2:** Incidence and prevalence of Crohn’s-related RVFs and AVFs: key findings and commentary (*n* = 3 studies)

Author, year	Study/base population	Incidence	Prevalence
Göttgens, 2017 [27]	All adult patients with CD in the IBD South Limburg cohort. Since 1991, this cohort has included incident adult IBD cases in the South Limburg area of the Netherlands Represents > 93% of all eligible patients in the region Mean (SD) age at CD diagnosis = 37.7 (15.9) years *n* = 1162 patients with CD; 728 female Netherlands (CD diagnosis during 1991–2011, follow-up until 2014)	*Overall cumulative probability of developing RVF among female patients with CD*: 0.7% after 1 year 1.7% after 5 years 3.1% after 10 years *Cumulative 10-year probability of developing RVF among female patients with CD*: 1.7% for patients diagnosed with CD during 1999–2011 5.7% for patients diagnosed with CD during 1991–1998	2.3% (17/728; calculated value) among female patients with CD
Schwartz, 2019 [25]	Cases of CD with codes for fistulizing disease (Truven Health MarketScan database) Age not reported *n* = 73,878 (95% CI: 72,203–75,553) for 2014 *n* = 75,666 (95% CI: 73,950–77,382) for 2017 USA (data up to 2014)	Not reported	2014 prevalence = 6064 (95% CI: 5656–6472) 2017 projected prevalence = 6211 (95% CI: 5793–6629)
Park, 2019 [21]	Patients with a CD diagnosis (Rochester Epidemiology Project medical records linkage system; health records of the residents of Olmsted County from Mayo Medical Center and Olmsted Medical Center) Pediatric: 14.3%^a^ (59/414) Adult: 85.7%^a^ (355/414) *n* = 414 patients with CD USA (CD diagnosis 1970–2010. Records reviewed until June 30, 2016)	Not reported	3.1% (13/414) of patients diagnosed with CD between 1970 and 2010 had ≥ 1 RVF or AVF episode, January 1, 1970–June 30, 2016

*AVF* anovaginal fistula*, CD* Crohn’s disease*, CI* confidence interval*, IBD* inflammatory bowel disease*, RVF* rectovaginal fistula, *SD* standard deviation

^a^Calculated value

Schwartz et al. (2019) conducted their database analysis to support findings from an SLR. Their SLR reported only one study which the authors used to estimate the prevalence of patients with CD who had 1 (65.4%), 2 (19.2%), 3 (8.2%), 4 (4.5%), and 5 (2.7%) episodes of Crohn’s-related RVF. The median duration of fistula episodes for patients with 1, 2, 3, 4, and 5 episodes was 2.2, 7.1, 12.1, 17.1, and 22.0 years, respectively. The weighted average of medians of the duration of fistulizing CD was 5.1 years [25].

A study using data from the Rochester Epidemiology Project found that 3.1% of patients (13/414) diagnosed with CD between 1970 and 2010 had at least one RVF or AVF episode [21]. The South Limburg study estimated the overall cumulative probability of developing an RVF among female patients as 0.7% after 1 year, 1.7% after 5 years, and 3.1% after 10 years from CD diagnosis. The cumulative 10-year probability of developing an RVF among female patients with CD was 1.7% for patients diagnosed with CD between 1999 and 2011 (down from 5.7% for diagnosis between 1991 and 1998, which is prior to the introduction of anti-tumor necrosis factor agents [anti-TNFs]) [27].

No studies of AVF incidence or prevalence were identified. However, it should be noted that the terminology around fistula classification is not completely standardized. Of specific relevance to the current SLR is the interchangeable use of the terms AVF and ‘low RVF’ [28]. Studies do not always specify whether the RVFs described in their results include ‘low RVF’ (i.e., AVF).

### Treatment patterns

Treatment patterns broadly refers to surgical procedures, medications, and conservative treatment including dietary modifications. It is known that RVF and AVF require substantial treatment; however, our systematic review did not identify any population-based studies addressing treatment patterns in these conditions. The current SLR includes 12 (non-population-based) studies that address treatment patterns by fistula type.

The sheer number of preceding interventions and repeat interventions per patient described in the included studies, along with ineffective treatments leading patients to try alternative treatments and procedures, are indicative of a high burden of disease. It is important to note that many of the studies identified through this SLR are clinic or hospital based and provide a snapshot of treatment patterns at that institution at the time of study. Similarly, clinic and hospital studies generally have limited numbers of patients and aim to compare one intervention versus another intervention, rather than providing a broad picture of treatment patterns for all patients with the condition of interest. While the focus of this article is the patient burden of RVF/AVF, the underlying burden of CD management including medications and surgeries is substantial.

Seven of 12 treatment pattern studies reported that patients had or required additional procedures before and/or after the intervention of interest, demonstrating a substantial treatment burden. It was frequently reported that patients had prior surgeries, such as anorectal surgery and fistula repair. For example, in a hospital-based study of 51 consecutive patients with CD who were undergoing treatment for RVF during 1998–2005, 40% of patients had previous anorectal surgery for CD. The median number of previous RVF surgical repairs in the group was 2–3, depending on the intervention group [15].

Six studies reported patients having other prior surgical interventions by fistula type, although some studies did not indicate whether the procedures were for the treatment of fistulas or the underlying CD. Procedures included seton drainage, diverting stoma creation, fistula plug, flap repair, fistulotomy, fistulectomy, fibrin glue, and sphincteroplasty [14–16, 19, 23, 26]. It should be noted that there is no single standard surgery for patients with AVF/RVF. Surgery of choice is dependent on location of fistula, severity, prior surgeries, degree of incontinence, and the surgeon’s clinical assessment and views on specific techniques (see Table 3).

**Table 3:** Studies providing information on treatment patterns (*n* = 12 studies)

Author, year	Baseline operations	Distribution of surgeries of interest	Surgery for failures/recurrence during follow-up	Immunosuppressive agents	Antibacterial agents
Corte, 2015 [9]	Not reported by fistula type	160 procedures in 34 patients with CD-related RVF Specific number for each procedure not reported by fistula type	Not reported by fistula type	*Pre-operative*: Investigators routinely propose anti-TNF therapy prior to surgery in patients with CD *Post-operative*: Not reported	*Pre-operative*: Not reported *Post-operative*: Not reported
El-Gazzaz, 2010 [14]	*Overall (n = 65)* Seton: 32.3%^a^ (21/65) Stoma: 60.0%^a^ (39/65) *Healed group (n = 30)* Seton: 40.0%^a^ (12/30) Stoma: 66.7%^a^ (20/30) *Unhealed group (n = 35)* Seton: 25.7%^a^ (9/35) Stoma: 54.3%^a^ (19/35)	*Overall* Mucosal advancement flaps: 72.3% (47/65) Episioproctotomy: 12.3% (8/65) Proctectomy and pull-through procedure with coloanal anastomosis: 10.8% (7/65) Fibrin glue: 3.1% (2/65) Fistula plug placement: 1.5% (1/65) 27.7%^a^ (18/65) of patients received > 3 repairs Median (range) number of repairs: Healed group: 2 (1–5) Unhealed group: 2 (1–8) *p* = 0.5 Median (IQR) interval from last repair to current, months: Healed group: 7.6 (4.1–11.1) Unhealed group: 9.7 (4.9–41.5) *p* = 0.1 Median (IQR) interval from seton to current repair, months: Healed group: 7.3 (5–8.9) Unhealed group: 4.2 (3.6–8.2) *p* = 0.5 Median (IQR) interval from stoma to current repair, months: Healed group: 5.7 (0.6–7.8) Unhealed group: 8 (0.9–22.9) *p* = 0.1	Not reported	*Pre-operative*: Immunomodulator use (infliximab, adalimumab, 6-mercaptopurine, and azathioprine within the 3 months prior to surgery): 40.0%^a^ (26/65) Steroids: 30.8%^a^ (20/65) *Post-operative*: Not reported	*Pre-operative:* Not reported *Post-operative:* Not reported
Gaertner, 2011 [15]	1. Previously received medical therapy: 94%^a^ (48/51) 2. Among patients who received surgery only (*n* = 25): - Previous RVF surgical repairs (median): 3 - Previous bowel resection for CD: 56% (14^a^/25) - Previous anorectal surgery for CD: 40% (10^a^/25) 3. Among patients who received surgery + infliximab (*n* = 26): - Previous RVF surgical repairs (median): 2 - Previous bowel resection for CD: 42% (11^a^/26) - Previous anorectal surgery for CD: 50% (13^a^/26) 4. Pre-operative fecal diversion: 19.6% (10/51) - 7 had undergone ileostomy - 3 had undergone colostomy	1. Total (*n* = 51 patients, 65 procedures): 54%^a^ (35/65) seton drainage 12%^a^ (8/65) advancement flap 12%^a^ (8/65) fibrin glue injection 9%^a^ (6/65) transperineal repair 6%^a^ (4/65) collagen plug placement 6%^a^ (4/65) bulbocavernosus (Martius) flap 2. In the surgery only group (*n* = 25 patients, 30 procedures): 60% (18/30) seton drainage 7%^a^ (2/30) advancement flap 20% (6/30) fibrin glue injection 13%^a^ (4/30) transperineal repair 0% (0/30) collagen plug placement 0% (0/30 bulbocavernosus (Martius) flap 3. In the surgery + infliximab group (*n* = 26 patients, 35 procedures): 49% (17/35) seton drainage 17% (6/35) advancement flap 6% (2/35) fibrin glue injection 6% (2/35) transperineal repair 11% (4/35) collagen plug placement 11% (4/35) bulbocavernosus (Martius) flap Note: a patient might have received > 1 surgery	Of the 9 patients who did not heal: - Seton insertion: 33%^a^ (3/9) - No seton insertion: 67%^a^ (6/9) 27% (14/51) patients eventually required proctectomy (*n* = 9 treated by surgery alone and *n* = 5 treated by surgery + infliximab)	*Pre-operative:* 1. In the surgery + infliximab group (*n* = 26): 100% received preoperative infliximab of 5 mg/kg at 0, 2, and 6 weeks (mean of 3.6 [range, 3– 6] infusions) and were also taking 6-mercaptopurine or azathioprine 2. In the surgery only group (who did not receive infliximab) (*n* = 25): 23% (5/25^c^) 5-ASA derivative 9% (2/25^c^) azulfidine 64% (14/25^c^) prednisone 9% (2/25^c^) azathioprine 5% (1/25^c^) methotrexate 50% (11/25^c^) 6-mercaptopurine *Post-operative:* Not reported	*Pre-operative:* In the operation only group (who did not receive infliximab) (*n* = 25), as reported by the authors: 73% (*n* = 16) metronidazole 50% (*n* = 11) ciprofloxacin Peri-operative short-course antibiotics were given to 78.4%^a^ (40/51) patients; assumed prophylactic *Post-operative:* Not reported
Göttgens, 2017 [27]	Not reported by fistula type	Not reported by fistula type	Not reported by fistula type	Not reported with respect to surgery *Exposure to immunomodulator or anti-TNFα therapy at any time prior to diagnosis of RVF*: 1991–1998: 70% (7/10) 1999–2011: 60% (3/5)	*Pre-operative:* Not reported by fistula type *Post-operative:* Not reported by fistula type
Jarrar, 2011 [16]	All patients underwent initial seton drainage and then flap repair ≥ 6 weeks later Other prior operations not reported by fistula type	Transanal endorectal advancement flap repair: 100% Note: if the fistula track was long it was drained with a mushroom catheter that was removed 10 days later. If the track was short the external opening was opened widely	Crohn’s AVF: *n* = 7 received 2nd flap *n* = 3 received 3rd flap *n* = 1 diverted Cryptoglandular perianal: *n* = 7 received 2nd flap *n* = 6 received 3rd flap *n* = 0 diverted Cryptoglandular anovaginal: *n* = 1 received 2nd flap *n* = 2 received 3rd flap *n* = 0 diverted	*Pre-operative:* Not reported *Post-operative:* Not reported	*Pre-operative:* Stated within 24 h prior to surgery and specified as prophylactic *Post-operative:* - Intravenous antibiotics continued post-surgery until discharge; unclear whether prophylactic - Oral antibiotics prescribed for 1 week
Korsun, 2019^b^ [17]	Not reported by fistula type	Not reported by fistula type	GMT: 100%	*Peri-operative*^*d*^: RVFs (*n* = 22 patients, including a patient with pouch and RVF) - Short-chain fatty acid: 4.5%^a^ (1/22) - Enema: 4.5%^a^ (1/22) - Azathioprine: 22.7%^a^ (5/22) - Steroids: 22.7%^a^ (5/22) - Colifoam: 4.5%^a^ (1/22) - Mercaptopurine: 9.1%^a^ (2/22) - Adalimumab: 9.1%^a^ (2/22) - MTX: 4.5%^a^ (1/22) - Sulfasalazine: 9.1%^a^ (2/22) - None: 45.5%^a^ (10/22) AVFs (*n* = 2 patients) - Azathioprine, steroids, mesalazine foam: 50%^a^ (1/2) - None: 50%^a^ (1/2) *Medication before GMT:* RVFs (*n* = 22 patients, including a patient with pouch and RVF) - Steroids: 9.1%^a^ (2/22) - Azathioprine: 13.6%^a^ (3/22) - Adalimumab: 9.1%^a^ (2/22) - Mercaptopurine: 9.1%^a^ (2/22) - Infliximab: 4.5%^a^ (1/22) - Sulfasalazine: 4.5%^a^ (1/22) - Mesalazine: 4.5%^a^ (1/22) - None: 4.5%^a^ (1/22) - Unknown: 59.1%^a^ (13/22) AVFs (*n* = 2 patients) - Unknown: 100.0%^a^ (2/2) *Medication after GMT:* RVFs (*n* = 22 patients, including a patient with pouch and RVF) - Mesalazine foam: 4.5%^a^ (1/22) - Steroids: 27.3%^a^ (6/22) - Azathioprine: 27.3%^a^ (6/22) - Adalimumab: 9.1%^a^ (2/22) - Sulfasalazine suppository/ sulfasalazine: 9.1%^a^ (2/22) - MTX: 4.5%^a^ (1/22) - Golimumab: 4.5%^a^ (1/22) - Mercaptopurine: 4.5%^a^ (1/22) - Unknown: 27.3%^a^ (6/22) - None: 22.7%^a^ (5/22) AVFs (*n* = 2 patients) - None: 50.0%^a^ (1/2) - Azathioprine: 50.0%^a^ (1/2) ≤ 1 medication per patient	*Pre-operative:* 100% received antibiotic (cefuroxime und metronidazole) 24 h prior to surgery—specified as prophylactic *Post-operative:* Not reported
Manne, 2016^b^ [26]	Past RVF surgery: - Cases: 50% (8/16) - Controls: 43% (20/47)	Proportion of patients who underwent mucosal flap procedure: - Cases: 88% - Controls: 12% Proportion of patients who underwent seton: - Cases: 13% - Controls: 77% Note: numbers for calculation not reported	Not reported	*Pre-operative steroid use (timing prior to surgery unclear)* - Cases: 25% (4/16) - Controls: 21% (10/47) *Azathioprine/6-mercaptopurine use* - Cases: 6% (1/16) - Controls: 15% (7/47) *Biologic use:* - Cases: 44% (7/16) - Controls: 62% (29/47) *MTX use:* - Cases: 6% (1/16) - Controls: 6% (3/47)	*Pre-operative:* Not reported *Post-operative:* Not reported
Narang, 2016 [19]	1. Had a seton before undergoing an attempted definitive surgical procedure: - Yes: 43.4% (43/99) - No: 56.6% (56/99) 2. Had a diverting stoma at the time of surgical repair: - Yes: 36.3% (36/99) - No: 63.6% (63/99)	Transrectal approach with endorectal advancement flap: 59.5%^c^ (59/99) Transvaginal repair: 14.1%^c^ (14/99) Muscle interposition: 14.1%^c^ (14/99) Martius or groin flaps: 9.6%^c^ (9/99) GMT: 5.3%^c^ (5/99) Episioproctotomy: 6.4%^c^ (6/99) Overlapping sphincteroplasty: 3.2%^c^ (3/99) Fibrin glue placement: 2.1%^c^ (2/99) Biological plug insertion: 1.1%^c^ (1/99) Note: reported calculations could not be replicated	Not reported	*At baseline:* Steroids: 57.6%^a^ (57/99) Infliximab: 48.5%^a^ (48/99) Adalimumab: 20.2%^a^ (20/99) Azathioprine: 4.0%^a^ (4/99) 6-mercaptopurine: 4.0%^a^ (4/99) *Follow-up:* Not reported	*Pre-operative:* Not reported *Post-operative:* Not reported
Oakley, 2015 [20]	Not reported by fistula type	Patients with Crohn’s RVF: 20%^a^ (4/20) patients received initial expectant therapy 80%^a^ (16/20) patients received initial surgery	Not reported	*Pre-operative:* Not reported by fistula type *Post-operative:* Not reported by fistula type	*Pre-operative:* Not reported by fistula type *Post-operative:* Not reported by fistula type
Pinto, 2010 [22]	Not reported by fistula type	In the 45 patients with CD, 80 procedures were performed: - Endorectal advancement flap: 47.5% (38/80) - GMT: 7.5% (6/80) - Transvaginal repair: 3.8% (3/80) - Transperineal repair: 3.8% (3/80) - Others: 37.5% (30/80)	Not reported	*Pre-operative:* Not reported by fistula type *Post-operative:* Not reported	*Pre-operative:* Not reported *Post-operative:* Not reported
Sapci, 2019 [23]	1. Previous surgery to close fistula: 57.9%^a^ (11/19) 2. History of ≥ 2 surgeries to close fistula: 52.6% (10^a^/19)	Transanal advancement flap: 42.1% (8/19) Transanal repair with tissue interposition (Martius or gracilis flap): 15.8% (3/19) Episioproctotomy: 10.5% (2/19) Fistulotomy: 10.5% (2/19) Coloanal anastomosis: 10.5% (2/19) Fistula plug: 10.5% (2/19) Active smoker: 42.1% (8/19)	Not reported	*Pre-operative:* Not reported *Post-operative:* Not reported	*Pre-operative:* Not reported *Post-operative:* Not reported
Schloericke, 2017 [24]	Recurrent cases included, but exact numbers and previous treatments are unclear	Patients with CD received resective surgical treatment only: - Low anterior resection: *n* = 6 - Subtotal colectomy: *n* = 3 (all patients indicated for this surgery based on presence of toxic megacolon) - Proctectomy: *n* = 1 - Pelvic exenteration: *n* = 1 Note: total number of patients with CD = 15, but only 11 surgeries reported	Proctectomy was performed in 1 case of recurrent fistulas in CD that led to severe sepsis	Not reported	Not reported

*5-ASA* aminosalicylate, *AVF* anovaginal fistula, *CD* Crohn’s disease, *GMT* gracilis muscle transposition, *IQR* interquartile range, *MTX* methotrexate, *RVF* rectovaginal fistula, *TNF* tumor necrosis factor

^a^Calculated value

^b^Medication information provided from corresponding author via email

^c^Numbers and percentages are reported as they were provided in the original article

^d^Numbers provided via correspondence from author in response to request for clarification

Another important aspect of treatment burden is the need for additional surgeries following the interventions of interest in the published studies. For example, 14 of the 51 patients (27%) described in the study above [15] eventually required proctectomies. In another study, seven of 21 patients with AVF who underwent transanal endorectal advancement flap repair received a second flap, three received a third flap, and one was diverted [16].

In addition to the surgical and procedural burden, this SLR indicated that patients with RVF/AVF report a heavy medication burden for both CD and fistula. For example, 94% of patients (48/51) who underwent treatment for RVF during 1998–2005 had received previous medication therapy, though it is unclear whether this was therapy for CD or fistula, specifically [15]. Seven of the 12 studies reporting on treatment patterns provided details on prior use of medications to manage CD and/or fistula. In a hospital-based study of 65 women who underwent surgery to close a RVF, 40% (26/65) had taken immunomodulators and 30.8% (20/65) had taken steroids within 3 months prior to surgery [14]. Reported medications include anti-TNF biologics, corticosteroids, azathioprine, methotrexate, 6 mercaptopurine, and antibiotics [9, 14–17, 19, 26, 27] (Table 3).

In addition to these surgical and medical treatments, other treatments for CD and RVF/AVF may include conservative management techniques, such as local wound debridement, a low residue diet, and sitz baths, although the data for these approaches are not enumerated in the literature [20].

### Clinical outcomes

Eleven studies included data on clinical outcomes for treatments of RVF/AVF (Table 4). The variability in treatments, study design, and description of outcomes further demonstrates the complexity of the clinical situation. As with the classification of the fistula overall (e.g., AVF vs ‘low RVF’), investigators use varying terminology, with or without clear definitions, to describe outcomes of interest (e.g., healing, closure, response).

**Table 4:** Interventions and success and failure rates in published studies (*n* = 11 studies)

Author, year	RVF/AVF sample size	Intervention(s)	Median follow-up duration, months (range)	Key outcome definitions	Success and failure rates			Post-operative infection rates
Corte, 2015 [9]	79 RVFs	Conservative procedures: seton drainage, vaginal advancement flap, rectal advancement flap, diverting stoma only, fistula plug, fibrin glue Major procedures: GMT, biomesh interposition, standard CAA or CRA, delayed CAA, abdominoperineal excision	33.1 (4–190); success ascertained at 3 months	Success: absence of any vaginal discharge of feces, flatus, or mucous discharge during ≥ 3 months after the last procedure AND absence of stoma. Patients who underwent a stoma performed after RVF healing for a non-RVF-related condition were considered as success	Success rate: 14.4% (23/160) in RVFs with CD etiology (160 procedures among 34 patients with CD-related RVF)			Not reported
El-Gazzaz, 2010 [14]	65 RVFs	Advancement flap, coloanal anastomosis, episioproctomy, fibrin glue or plug	44.6 (IQR: 13.1–79.1)	Healing (closed RVF): all pre-operative symptoms attributable to the fistula resolved at the time of follow-up and no fistula detected by physical examination at the last office visit	Healing rate, by type of current surgery: Mucosal advancement flap: 42.6% (20/47) CAA: 57.1% (4/7) Episioproctotomy: 71.4% (5/8)^b^ Fibrin glue: 50.0% (1/2) Plug: 0% (0/1)			Not reported
Gaertner, 2011 [15]	51 RVFs	Operative treatment, operative treatment + infliximab	38.6 (mean); (3–204)	Completely healed: no clinical evidence of fistula Minimally symptomatic: seton placement with minimal drainage and/or infliximab dependence Failure: persistent or recurrent symptomatic fistula, diverting procedure or proctectomy		Surgery only (*n* = 25)	Surgery + infliximab (*n = *26)	Not reported
					Completely healed	24% (6/25)	46% (12/26)	
					Minimally symptomatic	20% (5/25)	15% (4/26)	
					Healing rates: ‘completely healed’ + ‘minimally symptomatic’	44% (11/25)	62% (16/26)	
					Fistula closure	Not reported	54%^a^ (14/26)	
					Healing rates by operative approach (numbers for calculation not reported)			
						Surgery only (*n* = 25)	Surgery + infliximab (*n* = 26)	
					Transperianal repair (*n* = 6)	100%	50%	
					Seton drainage (*n* = 35)	33%	65%	
					Advancement flap (*n* = 8)	50%	0%	
					Fibrin glue (*n* = 8)	0%	0%	
					Martius flap (*n* = 4)	NA	75%	
					Collagen plug (*n* = 4)	NA	50%	
Haennig, 2015 [10]	12 RVFs	Seton drainage and associated treatment, infliximab, external drainage, fibrin glue, advancement flap, fistulotomy Other treatments (external drainage + infliximab, fistulotomy + infliximab, advancement flap + infliximab, infliximab [monotherapy], external drainage, bowel diversion)	64 (2–263)	Interval to closure: closure not defined	RVF: time interval to closure = 30.6 months vs 12 months for anal fistulas, *p* = 0.02 RVF not significantly correlated with relapse (*p* = 0.24)			Not reported
Jarrar, 2011 [16]	21 AVFs	Transanal endorectal advancement flap repair	Follow-up calls at 7 ± 3 years	Healing: not defined	Healing rate, after 1st flap: 41.7% (5/12) Healing rate, after 2nd flap: 42.9% (3/7) Healing rate, after 3rd flap: 66.7% (2/3) Healing rate, overall: 83.3% (10/12)			Not reported by fistula type
Korsun, 2019 [17]	21 RVFs 2 AVFs	GMT	47 (mean); (1–144)	Complete closure of fistula by 1st follow-up (~ 3 months post-operatively) without additional follow-up operations	Fistula closure rate: RVF: 71% (15^a^/21); including 1 patient with an abscess after GMT without fistula proof AVF: 50% (1^a^/2) Stoma closure rate: RVF: 55% (numerator unclear); 1 patient operated without stoma and 1 patient opting against stoma closure after fistula closure AVF: 50% (1^a^/2)			4.8%^a^ (1/21) patient with RVF had an abscess after the surgery without fistula proof
Milito, 2019 [18]	43 RVFs	Surgical approaches included drainage and seton, rectal advancement flap, vaginal advancement flap, transperineal approach using porcine dermal matrix, and Martius flap	18	Complete healing, healing rate and failure rate: not defined	Median time to ‘complete healing’: 6 months (range: 2–11 months) Healing rate: 81% (numbers for calculation not reported) Failure rate: 19% (numbers for calculation not reported)			Not reported
Narang, 2016 [19]	99 RVFs	Episioproctotomy, muscle interposition (including GMT and Martius flap), placement of biological plug and fibrin glue, rectal-advancement flap, sphincteroplasty, and transvaginal repair	39.1 (mean) ± 52.2 (SD)	Healing: not defined Failure to heal: persistence of symptoms that were compatible with the initial symptoms before surgical repair or current fecal drainage through the vagina	Overall healing: 63.7% (63/99)^b^ Healing in patients with prior seton: 55.8% (24/43) Healing in patients with prior stoma: 52.8% (19/36) Healing in patients with systemic steroid treatment within 30 days of surgery: 61.4% (35/57) Healing in patients with biologic therapy within 30 days of surgery: 63.2% (43/68)* *Note: numerator does not match the total healing count for infliximab and adalimumab, below Healing in patients with CD and obstetric injury: 74.0% (26/35)^b^ Healing in patients with steroids within 30 days of surgery: 61.4% (35/57) Healing in patients with infliximab within 30 days of surgery: 47.9% (23/48) Healing in patients with adalimumab within 30 days of surgery: 55.0% (11/20)			1 patient (1%^a^, 1/99) had urinary tract infection < 30 days after surgery
Pinto, 2010 [22]	45 of 125 RVFs were CD related	Endorectal advancement flap, GMT, transvaginal approach, transperineal approach	16.3 (mean)	Success: not defined Recurrence: persistence of symptoms compatible with the initial complaints and confirmed by physical examination or supplemental studies	Initial success rate: 44.2% (34/77 procedures) Recurrence rate: 55.8% (43/77 procedures) Eventual success rate (those who healed either initially or after recurrence): 78% (numbers for calculation not reported) after an average of 1.8 procedures			Not reported by fistula type
Sapci, 2019 [23]	19 RVFs	Transanal advancement flap, transanal repair with tissue interposition (Martius or gracilis flap), episioproctotomy, fistulotomy, CAA, fistula plug	29.6 (mean)	Success: no symptoms ≥ 6 months after definitive repair and/or stoma closure	Overall healing rate: 63% (12/19) Success rate in patients who received a biologic within 3 months of surgery: 50% (4/8) Successful closure by procedures: Transanal advancement flap: 50% (4/8) Transanal repair with tissue interposition (Martius or gracilis flap): 67% (2/3) Episioproctotomy: 100% (2/2) Fistulotomy: 100% (2/2) CAA: 100% (2/2) Fistula plug: 0% (0/2) Active smoker: 75% (6/8) Patients with peri-operative diversion had higher rates of success compared with no diversion group (66% vs 57%,* p* = 1)—numbers for calculation not reported			Not reported
Schloericke, 2017 [24]	58 RVFs	Non-resective procedures (transrectal/transvaginal omentoplasty or closure) Resective procedures (low anterior resection, subtotal colectomy, proctectomy, pelvic exenteration, double-barrel sigmoidostomy)	13 (3–36)	Recurrence: not defined	Complicated recurrence due to development of multiple perianal fistulas with severe sepsis: 13.3% (2/15)			In 13.3%^a^ (2/15) patients with CD, recurrence was complicated because of the development of multiple perianal fistulas with severe sepsis which led to emergency abdominoperineal excision of the rectum in one patient

*AVF* anovaginal fistula, *CAA* coloanal anastomosis, *CD* Crohn’s disease, *CRA* colorectal anastomosis, *GMT* gracilis muscle transposition, *IQR* interquartile range, *RVF* rectovaginal fistula

^a^Calculated value

^b^Numbers and percentages are reported as they were provided in the original article

Nevertheless, most studies include some assessment of success of the surgical procedure. For example, Haennig et al. (2015) identified the median ‘interval to fistula closure’ after seton drainage and infliximab treatment in 12 patients with RVF as 30.6 months [10]. Milito et al. (2019) measured median time to ‘complete healing’ in 43 patients with RVF as 6 months (range: 2–11 months) [18]. Other studies measured the rate of closure or healing, some by surgical type and some across surgical types. Of the nine studies that reported healing/success/closure across multiple surgical types, rates varied from 14.4 to 81% [9, 18], with seven ranging between 50 and 75% [14–19, 23]. Some of the variation may be explained by differences in study design, population characteristics, and surgical types included.

‘Recurrence rates’ were specifically reported in two studies and ranged from 13 (complicated recurrence due to development of multiple perianal fistulas with severe sepsis) to 55.8% across multiple procedures [22, 24]. Five studies reported post-operative infection rates; however, two [16, 22] did not report rates by fistula type. In the three studies that did report RVF/AVF-specific rates, 1–13% of patients experienced a post-operative infection, including one abscess [17], one urinary tract infection [19], and two cases of severe sepsis [24]. Further complicating interpretation of these results is the variance in median or mean follow-up duration which ranged from 13 months [24] to 7 years [16].

### Patient-reported outcomes

One of the 16 included studies offered findings collected through PRO instruments. El-Gazzaz et al. (2010) analyzed quality of life (QoL) data from the 12-item Short-Form Health Survey, Fecal Incontinence Quality of Life (FIQL), and Female Sexual Function Index (FSFI) questionnaires administered at surgical follow-up visits. The authors report ‘modest’ scores in the PRO instruments, with no significant difference between healed and unhealed women. For example, patients’ mean scores on the FIQL ranged from 2.5 to 3.1 (out of 5, with lower scores indicating lower QoL) in each of the scored domains (lifestyle, coping, depression, and embarrassment). FSFI total scores averaged 17.3 ± 6.7 and 17.9 ± 9.4 (from a possible total of 36, with lower scores indicating worse functioning) in healed and unhealed women, respectively. The surveys showed no statistically significant differences in QoL or sexual function among healed versus unhealed patients, and the authors suggest this may be due to the underlying effects of CD regardless of its complications [14]. This study is limited by the questionnaire completion rate among patients (45%) and reporting bias(es) regarding potential reticence of patients to discuss the sensitive nature of sexual health topics. Although a logical component of the disease burden in this population and one that may be captured in studies of CD overall, little is known about sexual interest and satisfaction among women with RVF/AVF. Similarly, more studies are needed to determine overall QoL and other insights into the patient experience that could be captured uniquely through PRO instruments.

#### Healthcare resource utilization

None of the 16 studies reported HCRU among patients by fistula type. More studies are needed to determine the direct and indirect costs of RVF and AVF, particularly as they relate to healthcare visits, copays, prescriptions, ancillary care such as psychological support, missed days at work and/or school, and productivity loss.

## Discussion

CD-related RVF and AVF are rare and devastating complications of a life-altering and debilitating disease. The current systematic literature review highlights knowledge gaps regarding the disease burden for patients with CD-related RVF and AVF. Many women experience painful and embarrassing symptoms and must endure numerous medical and procedural interventions with limited hope for efficacy. Despite the immense disease burden experienced by these women, the literature of observational nature offers only a limited view of the treatment patterns, clinical outcomes, and PROs of CD-related RVF and AVF. After a systematic search, we found 16 studies that met the a priori criteria for inclusion and were qualitatively synthesized to characterize and quantify the global epidemiological burden of non-perianal CD-related RVF, and only one described AVF. Ten of the 16 studies were carried out in the USA; the six remaining studies were conducted in Europe.

We found that very few population-based epidemiology studies have been published; however, one Netherlands-based study estimates the prevalence of RVF to be 2.3% among female patients with CD. A notable gap in the literature is the lack of precise definitions and standardized use of terminology throughout studies. The addition of more robust, population-based studies and registry studies would contribute to the current knowledge gap around the epidemiology of RVF and AVF globally and could be a potential topic for further research.

We also found that treatment patterns vary across the literature and are not well documented. Many studies were conducted in single centers and are limited by small sample size, retrospective study designs, and other factors that affect generalizability to broader populations. However, it is clear from the studies identified in this SLR that patients with RVF and AVF undergo myriad procedures both operative and non-operative, and also carry a heavy medication burden. Importantly, women with RVF and AVF experience frequent recurrence and subsequently undergo repeat procedures with variable success. Similarly, the SLR did not report robust literature on the effectiveness of these treatments in alleviating fistula symptoms or on the use of conservative management strategies (e.g., sitz baths and dietary approaches). Overall, there is a lack of standardized definitions or criteria to assess remission status, thus making it difficult to compare and make generalizable claims.

The range of techniques and multidisciplinary approach to address RVF and AVF is a function of the conditions’ distinct etiologies and the progressive nature of the underlying [28]. The American Society of Colon and Rectal Surgeons (ASCRS) provides a set of guidelines for the care of patients with RVF (Table 5). The literature does not provide reliable information about the efficacy of surgical treatments, and there is no clear treatment pattern for women with these conditions. The need for more robust population-based research and studies evaluating clinical effectiveness and comparative effectiveness across multiple therapeutic approaches is highlighted by the lack of high-quality evidence to support ASCRS treatment guidelines.

**Table 5:** ASCRS treatment guidelines for RVF

Recommendation	Grade of recommendation
Non-operative management is recommended for the initial management of obstetrical rectovaginal fistula and may also be considered for other benign and minimally symptomatic fistulas	Weak, based on low-quality evidence, 2C
A draining seton may be required to facilitate resolution of acute inflammation or infection associated with rectovaginal fistulas	Strong, based on low-quality evidence, 1C
Endorectal advancement flap, with or without sphincteroplasty, is the procedure of choice for most simple rectovaginal fistulas	Strong, based on low-quality evidence, 1C
Episioproctotomy may be used to repair obstetrical or cryptoglandular rectovaginal fistulas associated with extensive anal sphincter damage	Strong, based on low-quality evidence, 1C
A gracilis muscle or bulbocavernosus muscle (Martius) flap is recommended for recurrent or otherwise complex rectovaginal fistula	Strong, based on low-quality evidence, 1C
High rectovaginal fistulas that result from complications of a colorectal anastomosis often require an abdominal approach for repair	Strong, based on low-quality evidence, 1C
Proctectomy with colon pull-through or coloanal anastomosis may be required to repair radiation-related and recurrent complex rectovaginal fistula	Weak, based on low-quality evidence, 2C

*ASCRS* American Society of Colon and Rectal Surgeons, *RVF* rectovaginal fistula

Source: Vogel et al. (2016) [28]

It is the study authors’ opinion that additional knowledge gaps could be filled via studies that include population-based data from the USA and other countries, evaluate optimal induction and maintenance therapy, and incorporate additional PROs relating to QoL (including instruments such as the Wexner Incontinence Score and the FSFI questionnaire) [29, 30]. A review of data published since the search date uncovered an additional study by Seifarth et al. [31], which found a healing rate of 31.3% in patients with Crohn’s-related RVF after advancement flap placement. Although this demonstrates that research is ongoing in this area, data remains limited. The literature would benefit from the long-term follow-up of a longitudinal cohort of women with Crohn’s-related RVF or AVF to provide insights into patient outcomes well beyond the studied interventions.

Additionally, beyond the surgeries discussed in this review, it is important to note that other treatment methods, such as stem cell transplantation, have shown some degree of efficacy. A recent meta-analysis of Crohn’s fistula patients treated with stem cell transplantation utilized data from 7 studies of RVF and found a healing rate of 27.2% [32].

This study has several notable strengths. This is the first systematic review to identify knowledge gaps regarding the disease burden for patients with CD-related RVF and AVF. To accomplish this, a complete and thorough literature search with explicit eligibility criteria was undertaken across several databases, with articles subsequently screened for inclusion in this review. Selection, data extraction, and adjudication of risk of bias were done by two independent reviewers. Additional strengths of the current SLR include its compliance with established guidelines for SLRs, including the use of a pre-specified protocol and search criteria. The protocol was registered with PROSPERO to promote transparency and allow for future replication or updates. There are also a few limitation that should be noted. The SLR is limited by the overall lack of published data on Crohn’s-related RVF and AVF. Although the current search was designed to capture a wide range of literature, it is limited to recent publications in the last 10 years, and publications in English. Consequently, the resulting SLR might not be representative of the full body of published literature. Many of the studies were clinic-based, with short follow-up periods, and/or had small sample sizes, all of which limit generalizability. Furthermore, information on this topic area is only available where studies are published, so there is limited generalizability to populations where no data have been reported. Also, publication bias may have impacted the pool of available studies. Lastly, this SLR was designed to assess real-world evidence and therefore does not include data or follow-up from clinical trials. Although clinical trials include additional information on clinical outcomes and PROs, they are typically limited by short follow-up durations, strict inclusion criteria, and the inability to assess real-world outcomes. Despite these limitations, this SLR provides a unique summary of available data and highlights evidence gaps that can be addressed with further research.

## Conclusion

This systematic review reports a heavy patient burden among women with Crohn’s-related RVF and AVF in terms of both symptoms and medical and surgical treatment. Substantial gaps in knowledge surrounding Crohn’s-related RVF and AVF remain and more observational research is needed to support professional treatment guidelines with high-quality evidence.

## Supplementary Information

**Additional file 1**. Inclusion criteria for systematic review. Inclusion criteria followed the PICOTS framework.

## Data Availability

The datasets (original extraction sheets) used and/or analysed during the current study are available from the corresponding author on reasonable request.

## Abbreviations

ASCRS: American Society of Colon and Rectal Surgeons
AVF: Anovaginal fistula
CD: Crohn’s disease
CDAI: Crohn’s Disease Activity Index
EQ-5D: 5-Dimensional EuroQoL questionnaire
FIQL: Fecal Incontinence Quality of Life
FSFI: Female Sexual Function Index
HCRU: Healthcare resource utilization
IBDQ: Inflammatory Bowel Disease Questionnaire
PDAI: Perianal Disease Activity Index
PICOTS: Population, intervention, comparison, outcomes, time, and study design
PRISMA: Preferred Reporting Items for Systematic Reviews and Meta-Analyses
PRO: Patient-reported outcome
QoL: Quality of Life
ROBINS-I: Risk of Bias in Non-randomized Studies of Interventions
RVF: Rectovaginal fistula
SLR: Systematic literature review
