# Supplementary material for: A systematic review of the patient burden of Crohn’s disease-related rectovaginal and anovaginal fistulas
Source: BMC Gastroenterol. 2022 Jan 28;22:36. doi: 10.1186/s12876-021-02079-8 (PMC8796404; doi:10.1186/s12876-021-02079-8)
Supplement: Supplementary file 1 — Additional file 1. Inclusion criteria for systematic review. Inclusion criteria followed the PICOTS framework. [file 12876_2021_2079_MOESM1_ESM.docx]

Supplementary material

Supplementary Table 1 Inclusion criteria for systematic review

|  | **Inclusion criteria** |
| --- | --- |
| Population(s) | - Human patients with Crohn’s disease-related RVF or AVF - No restrictions regarding other demographic characteristics |
| Intervention(s) | - No restrictions for assessment of incidence/prevalence or HCRU/costs - Treatment patterns, ClinROs, and PROs focused on the following interventions: - Pharmacological: antibiotics, immunosuppressants, corticosteroids, prednisone, methotrexate, anti-tumor necrosis factor (infliximab, adalimumab, certolizumab pegol), monoclonal antibody (natalizumab, vedolizumab), interleukin antagonist (ustekinumab) - Surgical: fistulotomy, ligation of the intersphincteric fistula tract, cutting seton, fibrosing seton, drainage seton, sphincteric reconstruction, flap, ablation, fibrin glue, anal fistula plug, bioprosthetic plug, surgical reconstruction, sphincterotomy, sphincteroplasty, proctectomy, diversion, ostomy |
| Comparison(s) | - No restrictions on comparators for assessment of incidence/prevalence or HCRU/costs - Assessment of treatment patterns in studies that include the pharmacological or surgical interventions listed above - ClinROs and PROs in single-arm or comparator studies assessing the interventions listed above |
| Outcome(s) | - Incidence and prevalence - HCRU: outpatient physician visits, inpatient/hospital admissions, emergency room visits, laboratory tests, diagnostic and therapeutic tests, length of stay - Direct and indirect costs - Treatment patterns: proportion of patients utilizing pre-specified surgery or pharmacological treatment - Clinical outcomes: healing rate, success rate, response rate, recurrence rate, incontinence rate, fistula closure, clinical response, clinical remission - Specific PRO instruments of interest: CDAI, IBDQ, PDAI, RFIS, EQ-5D - Other PRO categories: pain, discharge/soiling, usage of pads, fecal incontinence, sexual intercourse/activity |
| Time | - No restriction on duration of illness or treatment - Publications within the past 10 years |
| Study design | - Observational studies: case-control studies, retrospective or prospective cohort studies/registries, cross-sectional studies - Pooled data excluded; however, studies identified in those reports would be reviewed as part of the manual search for inclusion. Other publications that are not original research, clinical trials, or case reports/series^a^ were excluded |
| Other-language | - English language |

*AVF* anovaginal fistula*, CDAI* Crohn’s Disease Activity Index, *ClinRO* clinical-reported outcome*, EQ-5D* 5‑dimensional EuroQoL questionnaire*, IBDQ* Inflammatory Bowel Disease Questionnaire*, HCRU* healthcare resource utilization*, PDAI* Perianal Disease Activity Index*, PRO* patient-reported outcome*, RFIS* Wexner Scale of Incontinence, Revised Fecal Incontinence Scale, *RVF* rectovaginal fistula

^a^Case series were included if they met pre-specified criteria for cohort design

Supplementary Table 2 PubMed search strategy (conducted on 25 March, 2020)

| **Row** | **Search concept** | **Terms** |
| --- | --- | --- |
| **Conditions** | | |
| #1 | **Condition 1**: Complex cryptoglandular fistulas and/or cryptoglandular fistulas | ((cryptoglandular[Title/Abstract] OR cryptogland*[Title/Abstract] OR horseshoe[Title/Abstract] OR branching[Title/Abstract]) AND  (“Fistula”[Mesh] OR “fistula”[Title/Abstract] OR fistul*[Title/Abstract])) OR  ((mid[Title/Abstract] OR high[Title/Abstract]) AND (transphincteric[Title/Abstract] OR trans-sphincteric[Title/Abstract] OR transsphincteric[Title/Abstract]) AND (“Fistula”[Mesh] OR “fistula”[Title/Abstract] OR fistul*[Title/Abstract])) |
| #2 | **Condition 2a**: Rectovaginal fistula | “Rectovaginal Fistula”[Mesh] OR  ((“rectovaginal”[Title/Abstract] OR “recto-vaginal”[Title/Abstract]) AND  (“Fistula”[Mesh] OR “fistula”[Title/Abstract] OR fistul*[Title/Abstract])) |
|  | **Condition 2b**: Anovaginal fistula | ((“anovaginal”[Title/Abstract] OR “ano-vaginal”[Title/Abstract]) AND  (“Fistula”[Mesh] OR “fistula”[Title/Abstract] OR fistul*[Title/Abstract])) |
|  | **Condition 2c**: Enterocutaneous fistula | ((“enterocutaneous”[Title/Abstract] OR “entero-cutaneous”[Title/Abstract]) AND  (“Fistula”[Mesh] OR “fistula”[Title/Abstract] OR fistul*[Title/Abstract])) |
|  | **Condition 2 combined**  2a OR 2b OR 2c | (((“Rectovaginal Fistula”[Mesh] OR ((“rectovaginal”[Title/Abstract] OR “recto-vaginal”[Title/Abstract]) AND (“Fistula”[Mesh] OR “fistula”[Title/Abstract] OR fistul*[Title/Abstract])))) OR (((“anovaginal”[Title/Abstract] OR “ano-vaginal”[Title/Abstract]) AND (“Fistula”[Mesh] OR “fistula”[Title/Abstract] OR fistul*[Title/Abstract])))) OR (((“enterocutaneous”[Title/Abstract] OR “entero-cutaneous”[Title/Abstract]) AND (“Fistula”[Mesh] OR “fistula”[Title/Abstract] OR fistul*[Title/Abstract]))) |
| #3 | Crohn’ Disease terms | “Crohn Disease”[Mesh] OR crohn*[Title/Abstract] |
| #4 | Fistula  (for title filter) | Fist*[Title] |
| **Real-world data terms** | | |
| #5 | Observational studies and real-world data | (“Cohort Studies”[Mesh] OR “Cross-Sectional Studies”[Mesh] OR “Longitudinal Studies”[Mesh] OR “Prospective Studies”[Mesh] OR “case-control study”[Mesh] OR “Registries”[Mesh] OR “Electronic Health Records”[Mesh] OR “Administrative Claims, Healthcare”[Mesh] OR “Observational Study” [Publication Type] OR  cohort[Title/Abstract] OR cohort*[Title/Abstract] OR cross-sectional[Title/Abstract] OR cross-section*[Title/Abstract] OR longitudinal[Title/Abstract] OR longitud*[Title/Abstract] OR prospective[Title/Abstract] OR prospect*[Title/Abstract] OR retrospective[Title/Abstract] OR observational[Title/Abstract] OR observation*[Title/Abstract] OR registry[Title/Abstract] OR “electronic medical record”[Title/Abstract] OR “medical record”[Title/Abstract] OR “electronic health record”[Title/Abstract] OR “health record”[Title/Abstract] OR (claims[Title/Abstract] OR “claims data”[Title/Abstract] OR “administrative claims”[Title/Abstract] OR “systematic review”[Title/Abstract] OR “systematic literature review”[Title/Abstract] OR “meta-analysis”[Title/Abstract]) |
| **Queries of interest** | | |
| #6 | Measures of occurrence/association | “Epidemiology”[Mesh] OR “Pharmacoepidemiology”[Mesh] OR  epidemiolog*[Title/Abstract] OR pharmacoepidemiolog*[Title/Abstract] OR  prevalence[Title/Abstract] OR incidence[Title/Abstract] OR risk[Title/Abstract] OR rate[Title/Abstract] OR rates[Title/Abstract] OR proportion[Title/Abstract] OR proportions[Title/Abstract] OR frequency[Title/Abstract] OR frequencies[Title/Abstract] |
| #7 | Health care resource utilization and costs | “Costs and Cost Analysis”[Mesh] OR “Economics”[Mesh] OR “Health Expenditures”[Mesh] OR “Cost of Illness”[Mesh] OR “Cost-Benefit Analysis”[Mesh] OR “Health Care Costs”[Mesh] OR “Direct Service Costs”[Mesh] OR “Hospital Costs”[Mesh] OR “Drug Costs”[Mesh] OR “Health Resources”[Mesh] OR “Resource Allocation”[Mesh] OR  “cost”[Title/Abstract] OR “costs”[Title/Abstract] OR “cost analysis”[Title/Abstract] OR “resource use”[Title/Abstract] OR “resource utilization”[Title/Abstract] OR expenditure[Title/Abstract] OR expenditures[Title/Abstract] OR economic[Title/Abstract] OR economics[Title/Abstract] |
| #8 | Treatment Patterns | “Immunosuppression”[Mesh] OR  “Immunosuppressive Agents”[Mesh] OR “Immunosuppressive Agents” [Pharmacological Action] OR “Anti-Bacterial Agents”[Mesh] OR “Anti-Bacterial Agents” [Pharmacological Action] OR “Antibodies, Monoclonal”[Mesh] OR “Surgical Procedures, Operative”[Mesh] OR “surgery” [Subheading] OR “Ostomy”[Mesh] OR  (immunosuppress*[Title/Abstract] OR antibiotic[Title/Abstract] OR antibiotics[Title/Abstract] OR corticosteroid*[Title/Abstract] OR prednisone[Title/Abstract] OR Methotrexate[Title/Abstract] OR anti-TNF[Title/Abstract] OR “anti-tumor necrosis factor”[Title/Abstract] OR infliximab[Title/Abstract] OR adalimumab[Title/Abstract] OR “certolizumab pegol”[Title/Abstract] OR certolizumab[Title/Abstract] OR “monoclonal antibody”[Title/Abstract] OR “monoclonal antibodies”[Title/Abstract] OR Natalizumab[Title/Abstract] OR vedolizumab[Title/Abstract] OR “interleukin antagonist”[Title/Abstract] OR “interleukin antagonists”[Title/Abstract] OR “interleukin inhibitor”[Title/Abstract] OR “interleukin inhibitors”[Title/Abstract] OR Ustekinumab[Title/Abstract] OR  cyclosporine[Title/Abstract] OR tacrolimus[Title/Abstract] OR  surgery[Title/Abstract] OR Fistulotomy[Title/Abstract] OR “ligation of the intersphincteric fistula tract”[Title/Abstract] OR LIFT[Title/Abstract] OR ligation*[Title/Abstract] OR seton[Title/Abstract] OR “cutting seton”[Title/Abstract] OR “fibrosing seton”[Title/Abstract] OR “drainage seton” OR “seton placement”[Title/Abstract] OR flap[Title/Abstract] OR “advancement flap”[Title/Abstract] OR “advancement flaps”[Title/Abstract] OR ablation[Title/Abstract] OR “fibrin glue”[Title/Abstract] OR “bioprosthetic plug”[Title/Abstract] OR “anal fistula plug”[Title/Abstract] OR plug[Title/Abstract] OR plugs[Title/Abstract] OR sealant [Title/Abstract] OR  sealants[Title/Abstract] OR “surgical reconstruction”[Title/Abstract] OR “sphincteric reconstruction”[Title/Abstract] OR sphincterotomy[Title/Abstract] OR sphincteroplasty[Title/Abstract] OR proctectomy[Title/Abstract] OR diversion[Title/Abstract] OR therapy[Title/Abstract] OR therapeutic*[Title/Abstract] OR pattern*[Title/Abstract] OR “treatment pattern” [Title/Abstract] OR “treatment patterns” [Title/Abstract] OR “ostomy”[Title/Abstract] OR ostomies[Title/Abstract]OR enterostomy[Title/Abstract] OR enterostomies[Title/Abstract] OR Cecostomy[Title/Abstract] OR Cecostomies[Title/Abstract] OR colostomy[Title/Abstract] OR colostomies[Title/Abstract] OR duodenostomy[Title/Abstract] OR  duodenostomies[Title/Abstract] OR ileostomy[Title/Abstract] OR ileostomies[Title/Abstract] OR jejunostomy[Title/Abstract] OR jejunostomies[Title/Abstract]) |
| #9 | Clinical outcomes | “Treatment Outcome”[Mesh] OR  ((“healing”[Title/Abstract] OR “success”[Title/Abstract] OR “response”[Title/Abstract] OR “recurrence”[Title/Abstract] OR “remission”[Title/Abstract] OR “incontinence”[Title/Abstract]) AND (“rate”[Title/Abstract] OR “frequency”[Title/Abstract] OR “proportion”[Title/Abstract])) OR  closure[Title/Abstract] OR “recurrence”[Title/Abstract] OR “recurrence risk”[Title/Abstract] OR “remission”[Title/Abstract] OR “remission risk”[Title/Abstract] OR “incontinence”[Title/Abstract] OR “incontinence risk”[Title/Abstract] OR “clinical response”[Title/Abstract] OR “clinical remission”[Title/Abstract] |
| #10 | Patient-reported outcomes | “Patient Reported Outcome Measures”[Mesh] OR “Fecal Incontinence”[Mesh] OR  (“patient reported outcomes”[Title/Abstract] OR “clinical outcome”[Title/Abstract] OR “Crohn’s Disease Activity Index”[Title/Abstract] OR CDAI[Title/Abstract] OR “IBD questionnaire”[Title/Abstract] OR “IBDQ”[Title/Abstract] OR “inflammatory bowel disease questionnaire”[Title/Abstract] OR “Patient reported outcome measures”[Title/Abstract] OR PROMs[Title/Abstract] OR “perianal disease activity index”[Title/Abstract] OR “PDAI”[Title/Abstract] OR “CDAI”[Title/Abstract] OR “anal pain”[Title/Abstract] OR pain[Title/Abstract] OR “fecal incontinence”[Title/Abstract] OR “faecal incontinence”[Title/Abstract] OR “bowel incontinence”[Title/Abstract] OR “fecal soiling”[Title/Abstract] OR “faecal soiling”[Title/Abstract] OR “leakage”[Title/Abstract] OR “discharge”[Title/Abstract] OR “soiling”[Title/Abstract] OR “soil”[Title/Abstract] OR “soiled”[Title/Abstract] OR “pads”[Title/Abstract] OR “EQ-5D”[Title/Abstract] OR “EuroQol”[Title/Abstract] OR “EuroQol-5D”[Title/Abstract] OR “intercourse”[Title/Abstract] OR “sexual activity”[Title/Abstract] OR “Wexner Scale of incontinence”[Title/Abstract] OR “Revised Fecal Incontinence Scale”[Title/Abstract] OR “Revised Faecal Incontinence Scale”[Title/Abstract] OR “RFIS”[Title/Abstract]) |
| **Combination searches** | | |
| #11 | Condition 2 + Crohn’s Disease | (#2a OR 2b OR 2c) AND #3 |
| #12 | Combined queries | #6 OR #7 OR #8 OR #9 OR #10 |
| **SEARCH RESULTS** | | |
| #13 | Condition 2 + Combined queries + real-world data + filters | #11 AND #12 AND #5 +English, human, 10 years filters |
| #14 | Condition 1 + combined queries + real-world data + filters | #1 AND #12 AND #5 +English, human, 10 years filters |
| #15 | Final search including fistula title search | (#13 OR #14) AND #4 |

Supplementary Table 3 Embase search strategy (conducted on 25 March, 2020)

| **Row** | **Search concept** | **Terms** |
| --- | --- | --- |
| **Conditions** | | |
| #1 | **Condition 1**: Complex cryptoglandular fistulas and/or cryptoglandular fistulas | ‘cryptoglandular fistula’:ab,ti,kw OR  ((cryptoglandular:ti,ab,kw OR horshoe:ab,ti,kw OR branching:ab,ti,kw) AND fistul*:ti,ab,kw) OR  ((mid:ab,ti,kw OR high:ab,ti,kw) AND (transphincteric:ab,ti,kw OR trans-sphincteric:ab,ti,kw OR transsphincteric:ab,ti,kw) AND fistul*:ti,ab,kw) |
| #2 | **Condition 2**:  Non perianal Crohn’s Disease fistula:  Rectovaginal fistulas, anovaginal Fistulas  Entero-cutaneous fistulas | ‘rectovaginal fistula’:ti,ab,kw OR ‘anovaginal fistula’:ti,ab,kw OR ‘enterocutaneous fistula’:ti,ab,kw OR  ((rectovaginal:ab,ti,kw OR recto-vaginal:ab,ti,kw OR anovaginal:ab,ti,kw OR ano-vaginal:ab,ti,kw OR enterocutaneous:ab,ti,kw OR entero-cutaneous:ab,ti,kw) AND fistul*:ab,ti,kw) |
| #3 | Crohn’s Disease terms | ‘crohn disease’:ab,ti,kw OR crohn*:ab,ti,kw |
| #4 | Fistula  (for title filter) | Fist*:ti |
| **Real-world data terms** | | |
| #5 | Observational studies and real-world data | ‘cohort analysis’:ab,ti,kw OR ‘cross-sectional study’:ab,ti,kw OR ‘cross sectional analysis’:ab,ti,kw OR ‘longitudinal study’:ab,ti,kw OR ‘prospective study’:ab,ti,kw OR ‘case control study’:ab,ti,kw OR ‘electronic medical record’:ab,ti,kw OR ‘electronic health record’:ab,ti,kw OR ‘administrative claims’:ab,ti,kw OR ‘observational study’:ab,ti,kw OR  ‘cohort’:ab,ti,kw OR cohort*:ab,ti,kw OR ‘cross-sectional’:ab,ti,kw OR ‘longitudinal’:ab,ti,kw OR ‘prospective’:ab,ti,kw OR ‘retrospective’:ab,ti,kw OR ‘observational’:ab,ti,kw OR ‘registry’:ab,ti,kw OR ‘register’:ab,ti,kw OR ‘registries’:ab,ti,kw OR ‘medical record’:ab,ti,kw OR ‘health record’:ab,ti,kw OR ‘medical record’:ab,ti,kw OR ‘claims data’:ab,ti,kw OR ‘administrative claims’:ab,ti,kw OR ‘claims’:ab,ti,kw OR ‘systematic review’:ab,ti,kw OR ‘systematic literature review’:ab,ti,kw OR ‘meta-analysis’:ab,ti,kw OR ‘meta analysis’:ab,ti,kw |
| **Queries of interest** | | |
| #6 | Measures of occurrence/association | epidemiolog*:ti,ab,kw OR pharmacoepidemiolog*:ti,ab,kw OR prevalence:ti,ab,kw OR incidence:ti,ab,kw OR rate:ti,ab,kw OR rates:ti,ab,kw OR risk:ti,ab,kw OR proportion:ti,ab,kw OR proportions:ti,ab,kw OR frequency:ti,ab,kw OR frequencies:ti,ab,kw |
| #7 | Health care resource utilization and costs | ‘resource utilization’:ti,ab,kw OR ‘resource use’:ti,ab,kw OR ‘resource allocation’:ti,ab,kw OR ‘health resources’:ti,ab,kw OR ‘health care utilization’:ti,ab,kw OR cost:ti,ab,kw OR ‘cost analysis’:ti,ab,kw OR economic:ti,ab,kw OR expenditures:ti,ab,kw OR ‘health care cost’:ti,ab,kw |
| #8 | Treatment patterns | ‘immunosuppressive agent’:ti,ab,kw OR ‘antibiotic drug’:ti,ab,kw OR  corticosteroid:ti,ab,kw OR prednisone:ti,ab,kw OR methotrexate:ti,ab,kw OR ‘anti tnf’:ti,ab,kw OR ‘tumor necrosis factor inhibitor’:ti,ab,kw OR infliximab:ti,ab,kw OR adalimumab:ti,ab,kw OR ‘certolizumab pegol’:ti,ab,kw OR ‘certolizumab’:ti,ab,kw OR ‘monoclonal antibody’:ti,ab,kw OR natalizumab:ti,ab,kw OR vedolizumab:ti,ab,kw OR ‘interleukin antagonist’ OR ‘interleukin inhibitor’:ti,ab,kw OR ustekinumab:ti,ab,kw OR  surgery:ti,ab,kw OR surgical:ti,ab,kw OR fistulotomy:ti,ab,kw OR sphincterotomy:ti,ab,kw OR sphincteroplasty:ti,ab,kw OR proctectomy:ti,ab,kw OR ligation:ti,ab,kw OR ‘ligation of the intersphincteric fistula tract’:ti,ab,kw OR lift:ti,ab,kw OR seton:ti,ab,kw OR ‘seton placement’:ti,ab,kw OR flap:ti,ab,kw OR ‘advancement flap’:ti,ab,kw OR ablation:ti,ab,kw OR ‘fibrin glue’:ti,ab,kw OR ‘bioprosthetic plug’:ti,ab,kw OR patterns:ti,ab,kw OR ‘treatment pattern’:ti,ab,kw OR ‘treatment patterns’:ti,ab,kw OR therapy:ti,ab,kw OR therapeutic:ti,ab,kw OR ‘cutting seton’:ti,ab,kw OR ‘fibrosing seton’:ti,ab,kw OR ‘drainage seton’:ti,ab,kw OR ‘sphincteric reconstruction’:ti,ab,kw OR ‘anal fistula plug’:ti,ab,kw OR sealant:ti,ab,kw OR ‘surgical reconstruction’:ti,ab,kw OR  ostomy:ti,ab,kw OR ostomies:ti,ab,kw OR enterostomy:ti,ab,kw OR enterostomies:ti,ab,kw OR cecostomy:ti,ab,kw OR cecostomies:ti,ab,kw OR colostomy:ti,ab,kw OR colostomies:ti,ab,kw OR duodenostomy:ti,ab,kw OR duodenostomies:ti,ab,kw OR ileostomy:ti,ab,kw OR ileostomies:ti,ab,kw OR jejunostomy:ti,ab,kw OR jejunostomies:ti,ab,kw |
| #9 | Clinical outcomes | ‘clinical outcome’:ti,ab,kw OR ‘clinical response’:ti,ab,kw OR remission:ti,ab,kw OR recurrence:ti,ab,kw OR closure:ti,ab,kw OR ‘incontinence’:ti,ab,kw OR  ‘healing rate’:ti,ab,kw OR ‘success rate’:ti,ab,kw OR  ‘response rate’:ti,ab,kw OR ‘recurrence rate’:ti,ab,kw OR ‘recurrence risk’:ti,ab,kw OR ‘remission rate’:ti,ab,kw OR ‘remission risk’:ti,ab,kw OR ‘incontinence rate’:ti,ab,kw OR ‘incontinence risk’:ti,ab,kw |
| #10 | Patient-reported outcomes | ‘patient-reported outcome’:ti,ab,kw OR ‘crohns disease patient-reported outcomes signs’:ti,ab,kw OR ‘cd pro’:ti,ab,kw OR ‘crohn disease activity index’:ti,ab,kw OR cdai:ti,ab,kw OR ‘ibd questionnaire’:ti,ab,kw OR ibdq:ti,ab,kw OR ‘inflammatory bowel disease questionnaire’:ti,ab,kw OR ‘patient reported outcome measure’:ti,ab,kw OR proms:ti,ab,kw OR ‘perianal disease activity index’:ti,ab,kw OR PDAI:ti,ab,kw OR discharge:ti,ab,kw OR soiling:ti,ab,kw OR soil:ti,ab,kw OR soiled:ti,ab,kw OR pads:ti,ab,kw OR ‘fecal incontinence’:ti,ab,kw OR ‘faecal incontinence’:ti,ab,kw OR ‘bowel incontinence’:ti,ab,kw OR ‘fecal soiling’:ti,ab,kw OR ‘faecal soiling’:ti,ab,kw OR ‘EQ-5D’:ti,ab,kw OR ‘EuroQol’:ti,ab,kw OR ‘EuroQol-5D’:ti,ab,kw OR intercourse:ti,ab,kw OR ‘sexual behavior’:ti,ab,kw OR ‘sexual activity’:ti,ab,kw OR ‘Wexner Scale of incontinence’:ti,ab,kw OR ‘Revised fecal incontinence scale’:ti,ab,kw OR ‘Revised faecal incontinence scale’:ti,ab,kw OR ‘RFIS’:ti,ab,kw OR ‘anal pain’:ti,ab,kw OR pain:ti,ab,kw OR ‘leakage’:ti,ab,kw |
| **Combination searches** | | |
| #11 | Condition 2 + Crohn’s Disease | #2 AND #3 |
| #12 | Combined queries | #6 OR #7 OR #8 OR #9 OR #10 |
| **SEARCH RESULTS** | | |
| #13 | Condition 2 + combined queries + real-world data + filters | #11 AND #12 AND #5 +English, human, 10 years filters |
| #14 | Condition 1 + combined queries + real-world data + filters | #1 AND #12 AND #5 +English, human, 10 years filters |
| #15 | Final search including fistula title search | (#13 OR #14) AND #4 |
